# Exploring the predictive values of CRP and lymphocytes in coronary artery disease based on a machine learning and Mendelian randomization

**DOI:** 10.3389/fcvm.2024.1442275

**Published:** 2024-09-09

**Authors:** Yuan Liu, Xin Yuan, Yu-Chan He, Zhong-Hai Bi, Si-Yao Li, Ye Li, Yan-Li Liu, Liu Miao

**Affiliations:** ^1^ Department of Cardiology, Liuzhou People’s Hospital, Affiliated of Guangxi Medical University, Liuzhou, Guangxi, China; ^2^ The Key Laboratory of Coronary Atherosclerotic Disease Prevention and Treatment of Liuzhou, Liuzhou, Guangxi, China

**Keywords:** coronary artery disease, lymphocytes, CRP, Mendelian randomization, retrospective analysis

## Abstract

**Purpose:**

To investigate the predictive value of leukocyte subsets and C-reactive protein (CRP) in coronary artery disease (CAD).

**Methods:**

We conducted a Mendelian randomization analysis (MR) on leukocyte subsets, C-reactive protein (CRP) and CAD, incorporating data from 68,624 patients who underwent coronary angiography from 2010 to 2022. After initial screening, clinical data from 46,664 patients were analyzed. Techniques employed included propensity score matching (PSM), logistic regression, lasso regression, and random forest algorithms (RF). Risk factors were assessed, and the sensitivity and specificity of the models were evaluated using receiver operating characteristic (ROC) curves. Additionally, survival analysis was conducted based on a 36-month follow-up period.

**Results:**

The inverse variance weight (IVW) analysis showed that basophil count (OR 0.92, 95% CI: 0.84–1.00, *P* = 0.048), CRP levels (OR 0.87, 95% CI: 0.73–1.00, *P* = 0.040), and lymphocyte count (OR 1.10, 95% CI: 1.04–1.16, *P* = 0.001) are significant risk factors for CAD. Using LASSO regression, logistic regression, and RF analysis, both CRP and lymphocyte counts were consistently identified as risk factors for CAD, prior to and following PSM. The ROC curve analysis indicated that the combination of lymphocyte and CRP levels after PSM achieves a higher diagnostic value (0.85). Survival analysis revealed that high lymphocyte counts and low CRP levels are associated with a decreased risk of Major Adverse Cardiovascular Events (MACE) (*P* < 0.001). Conversely, a higher CRP level combined with lymphocyte counts correlates with a poorer prognosis.

**Conclusion:**

There is a causal relationship between lymphocytes, CRP and CAD. The combined assessment of CRP and lymphocytes offers diagnostic value for CAD. Furthermore, high CRP levels coupled with low lymphocyte counts are associated with a poor prognosis.

## 1 Introduction

As the global population ages, the incidence of coronary artery disease (CAD) continues to increase. According to the World Health Organization's Global Burden of Ischemic Heart Disease Report from 1990 to 2019, approximately 9.14 million people died from CAD in 2019, while an estimated 197 million people worldwide were affected by CAD (1). Identifying risk factors closely associated with CAD is crucial for its prevention and treatment, as well as for reducing the social burden of the disease.

The development of CAD begins with endothelial damage, leading to lipid accumulation, atherosclerotic plaque formation, and ultimately, the progression of these plaques, causing narrowing or blockage of the coronary arteries (2). Low-density lipoprotein (LDL) plays a pivotal role in the formation of lipid plaques, and reducing LDL levels has been widely accepted in clinical guidelines (3). However, even among populations that have achieved LDL control targets, a significant residual risk of CAD persists, primarily due to other CAD risk factors, including systemic inflammation (4–6). Various inflammatory cells and mediators contribute to the formation and progression of lipid plaques. Studies assessing the role of inflammation in CAD risk have yielded inconsistent results (7–10). Epidemiological and multiple prospective cohort studies have demonstrated associations between C-reactive protein (CRP), leukocytes and their subgroups, and coronary artery disease (CAD) risk (11–20). Some research has even suggested a protective effect on the heart by reducing CRP levels in rats (21). Nonetheless, Mendelian randomization studies have not confirmed a causal relationship between CRP levels and CAD risk (22, 23). These conflicting findings necessitate a thorough investigation of the relationship between CRP, leukocytes, their subgroups, and CAD risk.

Two-Sample Mendelian Randomization (TSMR) is a frequently used method for analyzing disease risk factors (24, 25). Unlike traditional observational studies, TSMR is largely unaffected by confounding factors (26, 27). However, TSMR studies often lack subsequent clinical validation, potentially leading to conclusions that may contradict those of traditional randomized controlled trials and extensive observational studies. Initially, this study employed the TSMR approach to explore the association between leukocyte subsets, CRP, and CAD. It then utilized propensity score matching (PSM) on the study population, followed by logistic regression analysis, Lasso regression, and random forest algorithms to validate the TSMR findings. A clinical model was developed to assess the diagnostic efficiency of the TSMR method. To enhance the accuracy of the survival analysis, the study population was further stratified using ROC curves, thereby improving the reliability of the study conclusions.

## 2 Method

### 2.1 Mendelian randomization analysis

#### 2.1.1 Study design

The exposure factors were defined as leukocyte subsets and C-reactive protein (CRP) levels in the blood. Leukocyte subsets included eosinophil count, eosinophil percentage, monocyte count, neutrophil count, and lymphocyte count. The outcome variable was coronary artery disease (CAD).

#### 2.1.2 Data collection

Genome-wide association study (GWAS) data for eosinophil count (bbj-a-12), eosinophil percentage (ebi-a-GCST004600), monocyte count (ebi-a-GCST90002339), neutrophil count (ebi-a-GCST9000235), lymphocyte count (ukb-d-30120-irnt), CRP level (ieu-b-4764), and CAD (ebi-a-GCST005194) were obtained from the open GWAS project website (gwas.mrcieu.ac.uk). The characteristics of the leukocyte subsets and coronary heart disease datasets are shown in Table 1.

**Table 1:** Characteristics of white blood cell subgroups and coronary artery disease datasets.

Exposure	Cases/controls	Sample size	GWAS ID	population	Outcome	Cases/controls	Sample size	PMID	Population
Eosinophil count	—	108,953	bbj-a-12	East Asian	Coronary artery disease	34,541/261,984	296,525	29212778	European
Percentage of eosinophils	—	172,378	ebi-a-GCST004600	European	—	—	—	—	—
Monocyte count	—	13,471	ebi-a-GCST90002339	Mixed	—	—	—	—	—
Neutrophil count	—	78,744	ebi-a-GCST90002352	East Asian	—	—	—	—	—
Lymphocyte count	—	349,856	ukb-d-30120-irnt	European	—	—	—	—	—
C-reactive protein	—	61,308	ieu-b-4764	European	—	—	—	—	—

GWAS, genome-wide association study.

#### 2.1.3 Statistical analysis

In the two-sample Mendelian randomization (TSMR) analysis, instrumental variables (IVs) were employed for data analysis (26). The inclusion of IV genetic variants was required to satisfy the following three assumptions (27): (1) the IVs must be associated with leukocyte subset levels and CRP levels; (2) the IVs must not be influenced by confounding factors; and (3) the IVs must affect CAD exclusively through their association with leukocyte subset levels and CRP levels. To ensure a robust correlation between the IVs and leukocyte subset levels and CRP levels, a significance threshold of *P* < 5 × 10^−8^ was set, and single nucleotide polymorphisms (SNPs) meeting this criterion were selected as preliminary IVs. Additionally, the linkage disequilibrium coefficient r2r^2r2 was set to 0.001, and the region width was 10,000 kb to mitigate the influence of gene pleiotropy on the results. SNP information related to exposure was extracted from the CAD GWAS data. SNPs with high linkage were used to replace missing SNPs, and SNPs without replacement sites were excluded. The final IV dataset was obtained by combining data on leukocyte subset levels, CRP levels, and CAD. Four regression models were employed to assess the causal relationship between leukocyte subset levels, CRP levels, and CAD: MR-Egger regression, random effects inverse variance weighting (IVW), weighted median method, and weighted mode. Sensitivity analyses were conducted using heterogeneity tests, pleiotropy tests, and leave-one-out sensitivity tests. All analyses were performed using the TwoSampleMR package in R version 4.3.1, with a significance level (α) set at 0.05.

### 2.2 Case-control study

#### 2.2.1 Study design

**Figure 1:**
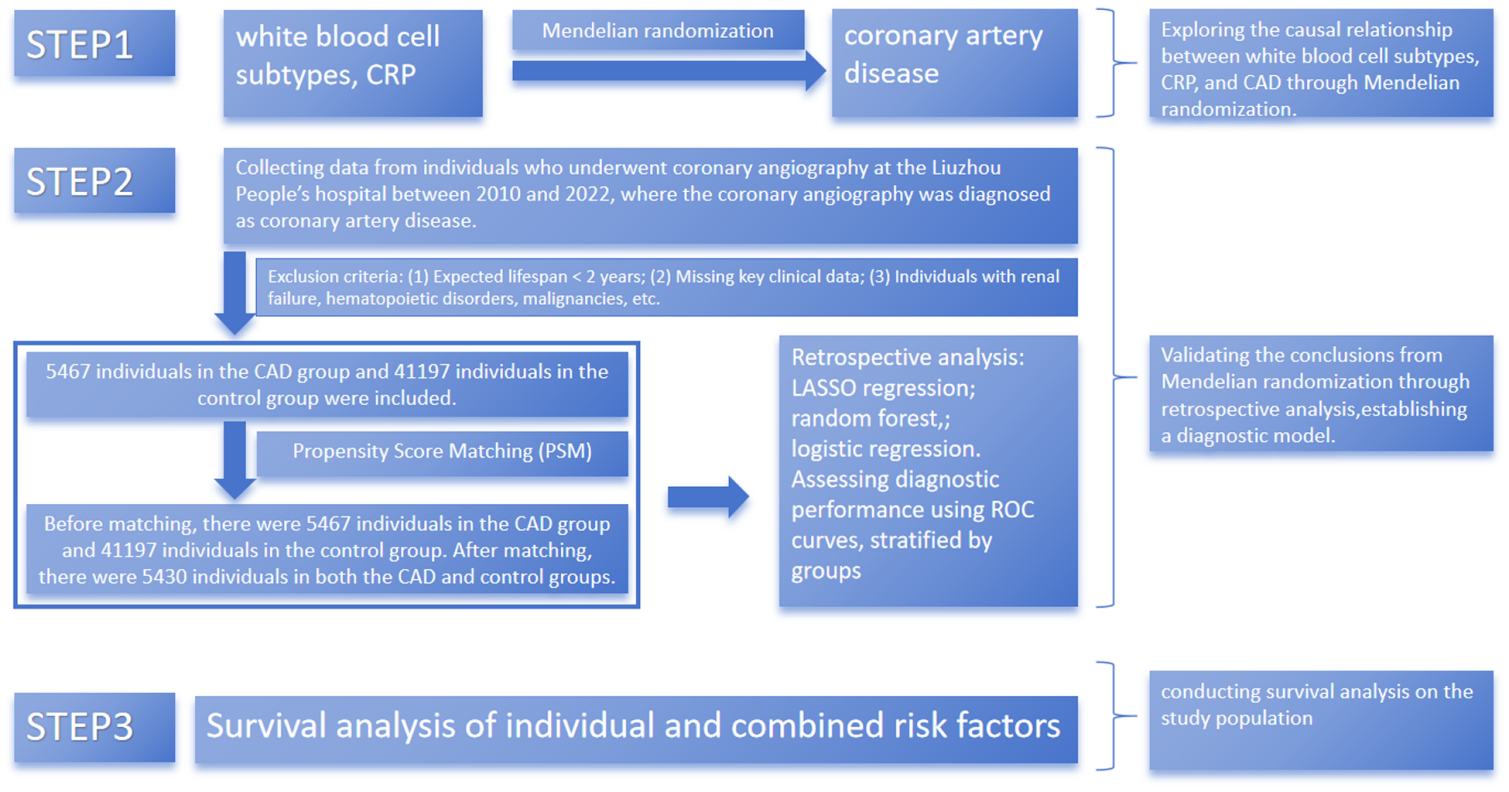
Process of recruiting patients for the study. CAD, coronary artery disease. CRP: C-reactive protein. Flowchart of patient recruitment and study design illustrating the selection and matching process to ensure balanced analysis groups.

This part of the study aimed to leverage large clinical sample data to validate the robustness of Mendelian randomization results through multiple analytical methods and to explore the predictive value of lymphocytes and C-reactive protein (CRP) for major adverse cardiovascular events (MACE). MACE was defined as cardiovascular death (death due to cardiovascular causes such as myocardial infarction, heart failure, or arrhythmia), nonfatal myocardial infarction (a heart attack that did not result in death), and nonfatal stroke (a cerebrovascular event that did not result in death). Figure 1 provides a flowchart of the study design.

#### 2.2.2 Data collection

Baseline clinical data were collected from patient medical records using spreadsheets. This study included clinical data from 68,624 patients who underwent coronary angiography in the Department of Cardiology at Liuzhou People's Hospital between 2010 and 2022. Inclusion criteria were as follows: patients who underwent coronary angiography and were diagnosed with coronary artery disease (CAD) with >50% stenosis of the main coronary artery branches. Baseline characteristics, including demographic information, clinical characteristics, and laboratory measurements, were collected. Key variables included left ventricular ejection fraction (LVEF) as an indicator of cardiac function and medication use [including angiotensin-converting enzyme inhibitors (ACEI), β-blocker, and antiplatelet drugs]. These factors were included to better account for variables affecting prognosis. Exclusion criteria were as follows: (1) life expectancy <2 years, (2) missing key clinical data, and (3) conditions such as renal failure, bone marrow hematopoietic dysfunction, or malignant tumors. After screening, 5,467 patients with coronary heart disease and 41,197 patients without coronary heart disease were included in the study. Patients were followed up regularly at 3, 6, 9, and 12 months after enrollment through telephone follow-up, readmission follow-up, and outpatient follow-up. The total follow-up period was 36 months. Propensity score matching (PSM) was performed using a caliper value of 0.02. This study involved a retrospective data analysis, and patient identity information was anonymized during the experimental design phase, ensuring that patient privacy was not compromised.

#### 2.2.3 Statistical analysis

For the clinical data of the study population, continuous data were expressed as mean ± standard deviation, and analysis of variance was used for intergroup comparisons. Categorical data were expressed as counts and ratios, with intergroup comparisons made using the chi-square test or Fisher's exact test. Propensity score matching (PSM) was employed to control potential confounders and create comparable patient groups for the CAD and non-CAD groups. LASSO regression was used to identify significant predictors of MACE, and the results were further validated using the random forest method. Multivariate logistic regression analysis was conducted to evaluate the predictive value of lymphocytes and CRP for MACE. For individuals meeting the diagnostic criteria for CAD, the receiver operating characteristic (ROC) curve was used to calculate the cutoff values of lymphocyte count and CRP level for grouping criteria, dividing the population into four groups: low lymphocyte count, high lymphocyte count, low CRP level, and high CRP level (28, 29). To evaluate the diagnostic performance of the models, we calculated the area under the ROC curve (AUC) for each model. The DeLong test was used to assess whether the differences in AUC between the models were statistically significant. Survival analysis was performed to assess the outcome of major adverse cardiovascular events (MACE).

## 3 Results

### 3.1 Mendelian randomization

The results are presented in Table 2. The MR-Egger method was employed to assess horizontal pleiotropy, with all groups demonstrating *P*-values greater than 0.05, indicating no evidence of horizontal pleiotropy. Heterogeneity among instrumental variables (IVs) was evaluated using Cochran's Q test (23). No heterogeneity was detected in monocyte count (*P* > 0.05). However, significant heterogeneity was observed among IVs for eosinophil count, eosinophil percentage, neutrophil count, CRP levels, and lymphocyte count, as indicated by Q statistics with *P*-values less than 0.05. Subsequent analyses were conducted using MR-Egger regression, random effects inverse variance weighting (IVW), the weighted median method, and the weighted mode, with particular emphasis on the IVW method. The IVW analysis revealed significant associations between coronary artery disease (CAD) and basophil count (IVW, OR 0.92, 95% CI: 0.84–1.00, *P* = 0.048), lymphocyte count (IVW, OR 1.10, 95% CI: 1.04–1.16, *P* = 0.001), and CRP levels (IVW, OR 0.87, 95% CI: 0.73–1.00, *P* = 0.040). These findings suggest causal relationships between CAD and eosinophil count, lymphocyte count, and CRP levels, as indicated by the IVW method (*P* < 0.05).

**Table 2:** Results of the mendelian randomization analysis.

Exposures	Outcome	Method	MR			*p*	Heterogeneity		Pleiotropy		*p*
			beta	SE	OR (95%CI)		Q	p	Intercept	SE	
Basophil count	CAD	MR egger	−0.063	0.110	0.94 (0.72–1.16)	0.576	43.815	0.002	−0.001	0.006	0.856
		Weighted median	−0.052	0.043	0.95 (0.86–1.03)	0.230					
		IVW	−0.081	0.041	0.92 (0.84–1.00)	0.048	43.890	0.002			
Eosinophil petcentage	CAD	IVW	−0.022	0.149	0.98 (0.69–1.27)	0.881	488.451	<0.05			
Monocyte count	CAD	MR egger	0.005	0.034	1.00 (0.94–1.07)	0.888	9.943	0.446	−0.004	0.005	0.433
		Weighted median	−0.006	0.017	0.99 (0.96–1.03)	0.725					
		IVW	−0.021	0.012	0.98 (0.96–1.00)	0.072	10.610	0.477			
Neutrophil count	CAD	MR egger	0.058	0.096	1.06 (0.87–1.25)	0.546	67.531	<0.05	−0.001	0.006	0.846
		Weighted median	0.053	0.036	1.05 (0.99–1.12)	0.133					
		IVW	0.041	0.033	1.04 (0.98–1.11)	0.216	67.617	<0.05			
C-reactive protein	CAD	MR egger	−0.407	0.141	0.67 (0.39–0.94)	0.008	178.849	<0.05	0.025	0.012	0.053
		Weighted median	−0.096	0.051	0.91 (0.81–1.01)	0.058					
		IVW	−0.141	0.069	0.87 (0.73–1.00)	0.040	212.759	<0.05			
Lymphocyte count	CAD	MR egger	0.007	0.071	1.01 (0.87–1.15)	0.924	1,074.044	<0.05	0.003	0.002	0.168
		Weighted median	0.031	0.029	1.03 (0.98–1.09)	0.285					
		IVW	0.095	0.030	1.10 (1.04–1.16)	0.001	1,081.179	<0.05			

CAD, coronary artery disease; SE standard error.

### 3.2 population characteristics

Propensity score matching (PSM) was employed to mitigate the influence of confounding variables and enhance the comparability between the experimental and control groups (30). A total of 46,664 participants were included in the study, comprising 5,467 patients with coronary artery disease (CAD) and 41,197 non-CAD individuals. Following PSM, each group consisted of 5,430 individuals. Before PSM, the CAD group exhibited significantly higher levels of age, systolic blood pressure (SBP), eosinophils, monocytes, homocysteine (Hcy), C-reactive protein (CRP), triglycerides (TG), and left ventricular ejection fraction (LVEF) compared to the control group (*P* < 0.05). Conversely, the CAD group had significantly lower levels of pulse pressure (PP), diastolic blood pressure (DBP), white blood cells (WBC), red blood cells (RBC), platelets (PLT), hematocrit (Hct), hemoglobin (Hb), CD4 + and CD8+ T cells, high-density lipoprotein cholesterol (HDL-C), low-density lipoprotein cholesterol (LDL-C), total cholesterol (TC), apolipoprotein B (ApoB), glycated hemoglobin (HbA1c), estimated glomerular filtration rate (eGFR), use of angiotensin-converting enzyme inhibitors (ACEI) or angiotensin II receptor blockers (ARBs), and beta-blockers (*P* < 0.001). After PSM, elevated WBC counts were observed only in the CAD group (*P* < 0.05), indicating a successful reduction of baseline differences between groups (see Table 3 for details).

### 3.3 Lasso regression

**Figure 2:**
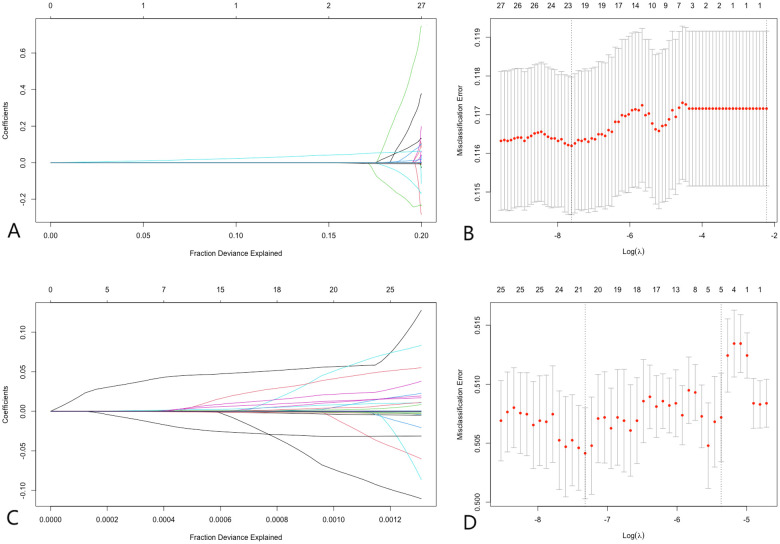
Lasso regression. **(A,B)** Demonstrate that before PSM, at the optimal lambda value (lambda = −7.61), 23 variables were retained, with variable importance ranking from highest to lowest as CRP, PLT, eosinophil count, percentage of eosinophils, and lymphocyte count, respectively. **(C,D)** Illustrate that after PSM, at the optimal lambda value (lambda = −7.32), 21 variables were retained, with variable importance ranking from highest to lowest as WBC, CRP, lymphocyte count, eosinophil count, and percentage of eosinophils, respectively. LASSO regression analysis identified CRP and lymphocyte count as significant predictors of CAD, demonstrating their importance in risk prediction.

Lasso regression was employed to address multicollinearity and facilitate feature selection (31). In the analysis of pre-matched data, lasso regression identified the most significant variables at the optimal lambda value (−7.61), ranking them in descending order as follows: C-reactive protein (CRP), platelets (PLT), eosinophil count, percentage of eosinophils, and lymphocyte count. Similarly, the analysis of post-matched data at the optimal lambda value (−7.32) ranked the variables as follows: white blood cell count (WBC), CRP, lymphocyte count, eosinophil count, and percentage of eosinophils. Notably, CRP, eosinophil count, percentage of eosinophils, and lymphocyte count were consistently identified as significant variables regardless of matching status. This consistency underscores the robustness of these variables in predicting coronary artery disease (CAD). Figure 2 illustrates the results of the lasso regression analysis.

### 3.4 Logistic regression

The dependent variable, coronary artery disease (CAD) diagnosis, was dichotomized using a binary categorical variable assignment method, with CAD coded as 1 and non-CAD as 0. A stepwise approach was subsequently applied to incorporate these variables into the logistic regression model. Before matching, the risk factors considered for CAD included age, gender, triglycerides (TG), monocyte count, glomerular filtration rate, total cholesterol (TC), body mass index (BMI), C-reactive protein (CRP), eosinophil percentage, platelets, hemoglobin (Hb), homocysteine (Hcy), high-density lipoprotein cholesterol (HDL-C), low-density lipoprotein cholesterol (LDL-C), pulse pressure (PP), apolipoprotein B (ApoB), and lymphocyte count. After matching, the risk factors for CAD were identified as Hcy, HDL-C, white blood cell count (WBC), age, Hb, BMI, glomerular filtration rate, CRP, lymphocyte count, and red blood cell count (RBC). CRP and lymphocyte count were consistently identified as significant risk factors regardless of matching status. Detailed results are provided in Tables 4, 5.

**Table 3:** General characteristics of patients before and after PSM matching.

Parameter	Before				After			
	CAD (*n* = 5,467)	Control (*n* = 41,197)	SMD	*P* value	CAD (*n* = 5,430)	Control (*n* = 5,430)	SMD	*P* value
Age	67.00 (13.00)	48.00 (17.48)	1.233	<0.001	66.92 (13.00)	67.06 (13.55)	0.011	0.583
Gender = male (%)	2,660 (48.65)	20,414 (49.55)	0.148	0.213	3,028 (55.76)	2,965 (54.60)	0.023	0.224
PP	59.46 (26.73)	64.02 (24.51)	0.178	<0.001	59.56 (26.67)	59.61 (27.21)	0.002	0.923
SBP	124.76 (37.58)	117.93 (31.80)	0.196	<0.001	124.83 (37.47)	125.25 (37.90)	0.011	0.561
DBP	63.44 (21.44)	67.46 (19.28)	0.197	<0.001	63.52 (21.38)	63.61 (21.65)	0.004	0.827
BMI	28.54 (9.68)	28.34 (7.72)	0.023	0.081	28.54 (9.68)	28.73 (8.16)	0.021	0.269
WBC	0.04 (0.55)	0.07 (0.70)	0.041	<0.05	0.04 (0.55)	0.02 (0.40)	0.036	<0.04
RBC	4.29 (1.16)	4.47 (1.13)	0.152	<0.001	4.29 (1.17)	4.29 (1.29)	<0.001	>0.999
PLT	221.13 (87.96)	240.99 (85.20)	0.229	<0.001	221.29 (87.91)	221.42 (89.17)	0.002	0.940
Hct	38.71 (10.34)	39.75 (9.94)	0.103	<0.001	38.69 (10.37)	38.62 (11.45)	0.006	0.738
Hb	13.06 (3.52)	13.47 (3.40)	0.118	<0.001	13.06 (3.53)	13.03 (3.88)	0.009	0.673
Basophil	0.04 (0.06)	0.04 (0.06)	0.043	>0.999	0.04 (0.06)	0.04 (0.09)	0.008	>0.999
Eosinophil	0.19 (0.20)	0.17 (0.18)	0.103	<0.001	0.19 (0.19)	0.19 (0.24)	0.011	>0.999
Lymphocyte	1.74 (2.35)	1.88 (2.12)	0.058	<0.001	1.73 (2.27)	1.78 (5.17)	0.013	0.514
Monocyte	0.51 (0.31)	0.47 (0.27)	0.121	<0.001	0.51 (0.30)	0.50 (0.33)	0.014	0.099
CD4	0.43 (22.77)	1.75 (41.21)	0.040	<0.05	0.43 (22.85)	0.03 (2.58)	0.024	0.200
CD8	0.34 (19.22)	1.44 (32.22)	0.041	<0.05	0.34 (19.28)	0.12 (8.83)	0.015	0.445
Fer	1.21 (28.81)	1.15 (16.59)	0.003	0.821	1.22 (28.91)	0.65 (13.94)	0.025	0.191
Hcy	1.14 (4.23)	0.85 (2.89)	0.080	<0.001	1.15 (4.24)	1.05 (3.99)	0.024	0.206
CRP	0.33 (0.92)	0.22 (0.62)	0.143	<0.001	0.32 (0.86)	0.31 (1.01)	0.016	0.579
HDL-C	0.11 (0.38)	0.13 (0.41)	0.039	<0.001	0.11 (0.38)	0.11 (0.39)	0.004	>0.999
LDL-C	1.03 (1.43)	1.19 (1.57)	0.102	<0.001	1.04 (1.43)	1.02 (1.46)	0.008	0.471
TG	1.52 (1.47)	1.46 (1.53)	0.038	<0.001	1.52 (1.47)	1.52 (2.13)	0.004	>0.999
TC	3.85 (2.10)	4.30 (2.05)	0.217	<0.001	3.86 (2.10)	3.86 (2.27)	0.001	>0.999
ApoB	0.27 (0.44)	0.29 (0.46)	0.042	<0.05	0.27 (0.44)	0.27 (0.44)	0.001	>0.999
HbA1c	0.03 (0.45)	0.05 (0.54)	0.038	<0.05	0.03 (0.45)	0.02 (0.36)	0.031	0.201
HBG	0.01 (0.30)	0.02 (0.37)	0.023	0.055	0.01 (0.30)	0.01 (0.26)	0.025	>0.999
Scr	0.68 (15.70)	0.76 (8.34)	0.007	0.558	0.68 (15.75)	0.34 (6.33)	0.028	0.140
eGFR	66.27 (30.13)	89.71 (31.80)	0.757	<0.001	66.51 (30.01)	66.83 (31.84)	0.010	0.590
LVEF (%)	55.2 (8.1)	54.1 (8.3)	0.13	<0.05	55.2 (8.1)	55.3 (8.0)	0.011	0.791
ACEI or ARB (%)	4,897 (89.5)	37,500 (91.0)	0.05	<0.05	4,897 (90.2)	4,885 (89.9)	0.014	0.661
β (%)	3,450 (63.1)	29,031 (70.4)	0.15	<0.05	3,450 (63.6)	3,420 (63.0)	0.010	0.783
Antiplatelet agent	4,905 (89.6)	12,350 (30.1)	1.33	<0.05	4,905 (90.2)	4,885 (89.9)	0.018	0.662

Chi-square test for gender, LVEF, ACEI or ARB, β, Antiplatelet Agent. SMD, Standardized Mean Difference; PP, pulse pressure; SBP, systolic blood pressure; DBP, diastolic blood pressure; BMI, body mass index; WBC, white blood cell; RBC, red blood cell; PLT, platelets; Hct, hematocrit; Hb, hemoglobin; CD4, CD4 T cell; CD8, CD8 T cell; Fer, ferritin; Hcy, homocysteine; CRP, c-reactive protein; HDL.C, high-density lipoprotein; LDL.C, low-density lipoprotein; TG, triglyceride; TC, total cholesterol; ApoB, apolipoprotein B; HbA1c, glycated hemoglobin; HBG, fasting blood glucose; Scr, serum creatinine; eGFR, estimated glomerular filtration rate; LVEF, Left Ventricular Ejection Fraction; ACEI or ARB, Angiotensin-Converting Enzyme Inhibitor or Angiotensin II Receptor Blocker; β, Beta-blockers.

**Table 4:** Logistic regression before PSM matching.

Parameter	B	S.E	*P* value	OR	2.5% CI	97.5% CI
PLT	−0.001	0.000	0.001	0.999	0.998	0.999
Hct	0.063	0.018	0.001	1.065	1.029	1.103
Hb	−0.178	0.044	0.001	0.837	0.768	0.913
Eosinophil	0.383	0.089	0.001	1.467	1.233	1.746
Monocyte	0.752	0.076	0.001	2.121	1.828	2.461
Hcy	0.042	0.008	0.001	1.042	1.026	1.059
CRP	0.136	0.019	0.001	1.146	1.103	1.19
HDL.C	−0.293	0.078	0.001	0.746	0.64	0.869
TG	0.107	0.012	0.001	1.113	1.088	1.138
TC	−0.168	0.012	0.001	0.845	0.825	0.866
Gender	−0.237	0.035	0.001	0.789	0.737	0.845
BMI	0.017	0.002	0.001	1.017	1.013	1.021
Age	0.063	0.001	0.001	1.065	1.062	1.068
eGFR	−0.007	0.001	0.001	0.993	0.991	0.994
ApoB	0.212	0.063	0.001	1.237	1.093	1.399
Lymphocyte	−0.013	0.009	0.013	0.987	0.971	1.004
PP	−0.002	0.001	0.021	0.998	0.996	1
LDL.C	−0.029	0.019	0.142	0.972	0.936	1.01
Scr	0.003	0.002	0.293	1.003	0.998	1.008
RBC	0.055	0.058	0.342	1.057	0.943	1.184
WBC	−0.072	0.100	0.468	0.930	0.765	1.131
Fer	0.001	0.001	0.568	1.001	0.998	1.003
SBP	0.000	0.001	0.593	1.000	0.998	1.001
CD8	−0.001	0.001	0.603	0.999	0.997	1.002
HbA1c	0.060	0.132	0.652	1.061	0.819	1.376
HBG	−0.021	0.065	0.748	0.979	0.861	1.113
CD4	0.000	0.001	0.805	1.000	0.998	1.002
Basophil	0.065	0.268	0.810	1.067	0.631	1.804
DBP	0.000	0.001	0.884	1.000	0.997	1.003

WBC, white blood cell; RBC, red blood cell; PLT, platelets; Hct, hematocrit; Hb, hemoglobin; CD4, CD4 T cell; CD8, CD8 T cell; Fer, ferritin; Hcy, homocysteine; CRP, c-reactive protein; HDL.C, high-density lipoprotein; LDL.C, low-density lipoprotein; TG, triglyceride; TC, total cholesterol; ApoB, apolipoprotein B; HbA1c, glycated hemoglobin; HBG, fasting blood glucose; BMI, body mass index; Scr, serum creatinine; eGFR, estimated glomerular iltration rate; PP, pulse pressure; SBP, systolic blood pressure; DBP, diastolic blood pressure.

**Table 5:** Logistic regression after PSM matching.

Parameter	B	S.E	*P* value	OR	2.50% CI	97.50% CI
Hcy	0.012	0.008	0.014	1.012	0.997	1.028
HDL.C	−0.124	0.085	0.015	0.883	0.745	1.042
(Intercept)	0.249	0.184	0.018	1.283	0.895	1.839
WBC	0.203	0.177	0.025	1.224	0.875	1.773
Age	−0.002	0.002	0.028	0.998	0.994	1.002
Hb	0.055	0.053	0.030	1.056	0.952	1.172
BMI	−0.002	0.002	0.032	0.998	0.993	1.002
eGFR	−0.001	0.001	0.036	0.999	0.997	1.001
CRP	0.018	0.021	0.040	1.018	0.977	1.062
HbA1c	−0.177	0.209	0.040	0.838	0.539	1.246
Lymphocyte	−0.005	0.007	0.042	0.995	0.977	1.006
RBC	−0.057	0.070	0.042	0.945	0.823	1.084
Gender	−0.031	0.043	0.047	0.970	0.892	1.054
Monocyte	0.058	0.086	0.049	1.060	0.896	1.254
LDL.C	0.015	0.024	0.053	1.015	0.968	1.065
HBG	0.051	0.091	0.058	1.052	0.884	1.275
Hct	−0.011	0.021	0.060	0.989	0.948	1.031
ApoB	−0.034	0.078	0.066	0.967	0.830	1.126
TG	−0.005	0.013	0.071	0.995	0.969	1.021
Basophil	0.097	0.286	0.074	1.101	0.632	1.981
Scr	0.001	0.003	0.084	1.001	0.995	1.009
Eosinophil	0.021	0.104	0.084	1.021	0.832	1.254
SBP	0.000	0.001	0.088	1.000	0.998	1.001
DBP	0.000	0.002	0.090	1.000	0.997	1.003
PLT	0.000	0.000	0.092	1.000	0.999	1.001
CD4	0.065	0.700	0.093	1.067	0.904	1.785
CD8	−0.036	0.427	0.093	0.965	0.705	1.067
Fer	0.000	0.001	0.095	1.000	0.997	1.003
PP	0.000	0.001	0.095	1.000	0.998	1.002
TC	0.001	0.015	0.097	1.001	0.972	1.030

WBC, white blood cell; RBC, red blood cell; PLT, platelets; Hct, hematocrit; Hb, hemoglobin; CD4, CD4 T cell; CD8, CD8 T cell; Fer, ferritin; Hcy, homocysteine; CRP, c-reactive protein; HDL.C, high-density lipoprotein; LDL.C, low-density lipoprotein; TG, triglyceride; TC, total cholesterol; ApoB, apolipoprotein B; HbA1c, glycated hemoglobin; HBG, fasting blood glucose; BMI, body mass index; Scr, serum creatinine; eGFR, estimated glomerular iltration rate; PP, pulse pressure; SBP, systolic blood pressure; DBP, diastolic blood pressure.

### 3.5 Random forest

**Figure 3:**
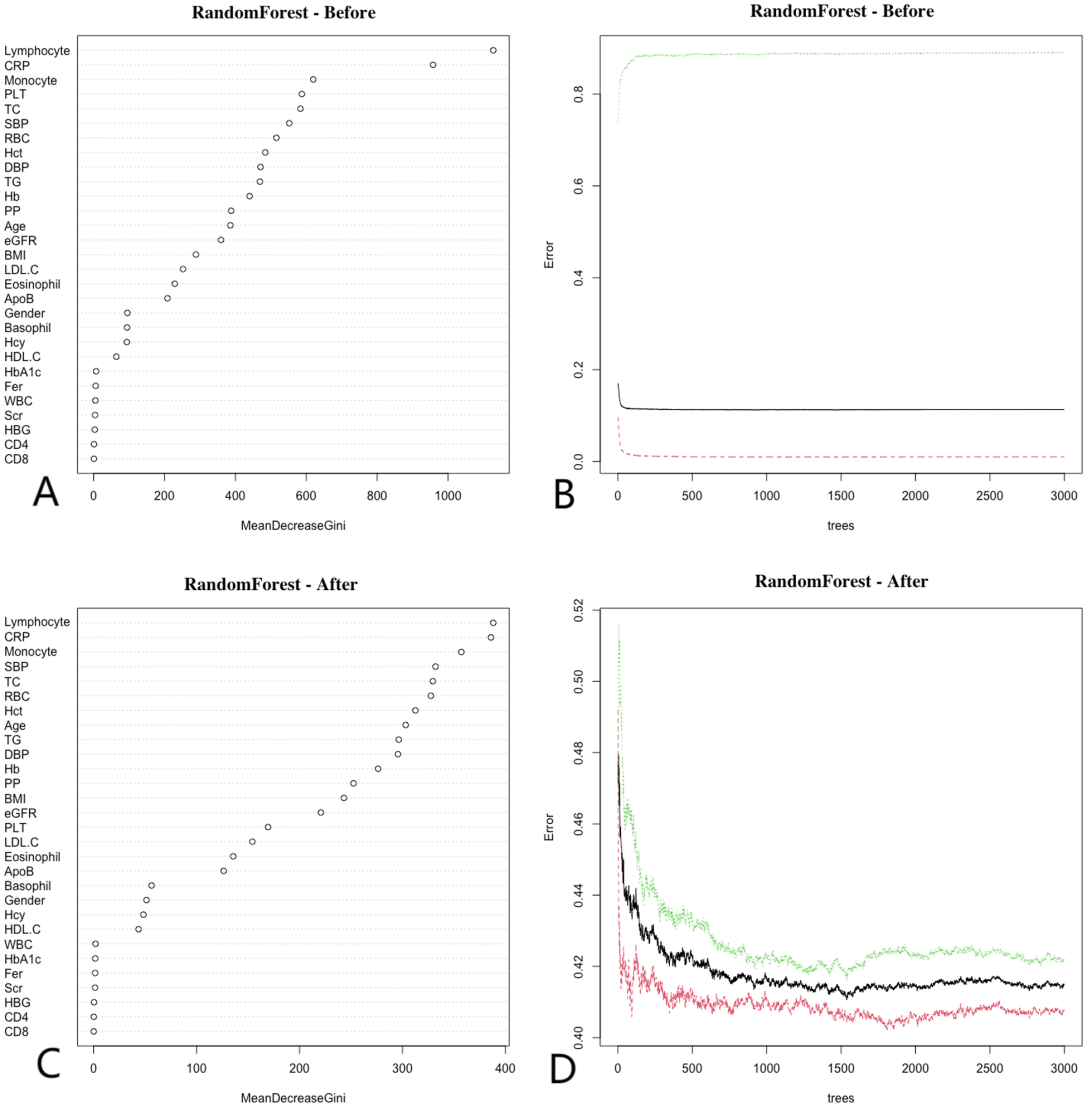
Random forest. Before matching, **(A)** show that the top three ranked variables were lymphocyte count, CRP, and monocyte count, **(B)** show that the error rate of the model stabilizes when the number of classification trees exceeds 500; After matching, **(C)** show that the top three ranked variables remained lymphocyte count, CRP, and monocyte count, **(D)** show that the error rate of the model stabilizes when the number of classification trees exceeds 1,000. Random forest analysis highlights key predictors of CAD, emphasizing the importance of CRP and lymphocyte count in the model.

The Random Forest (RF) algorithm determines the relative importance of each indicator by evaluating the average reduction in prediction accuracy (32). Figure 3 illustrates the importance rankings of risk factors for coronary artery disease (CAD), where higher values denote greater significance. RF analysis was performed on both pre-matched and post-matched data, with variables arranged in descending order of importance. Before matching, the top three variables were lymphocyte count, C-reactive protein (CRP), and monocyte count. After matching, these same top three variables—lymphocyte count, CRP, and monocyte count—remained the most significant. This consistency across matching statuses further emphasizes the critical role of these variables in predicting CAD.

### 3.6 Diagnostic efficacy

**Figure 4:**
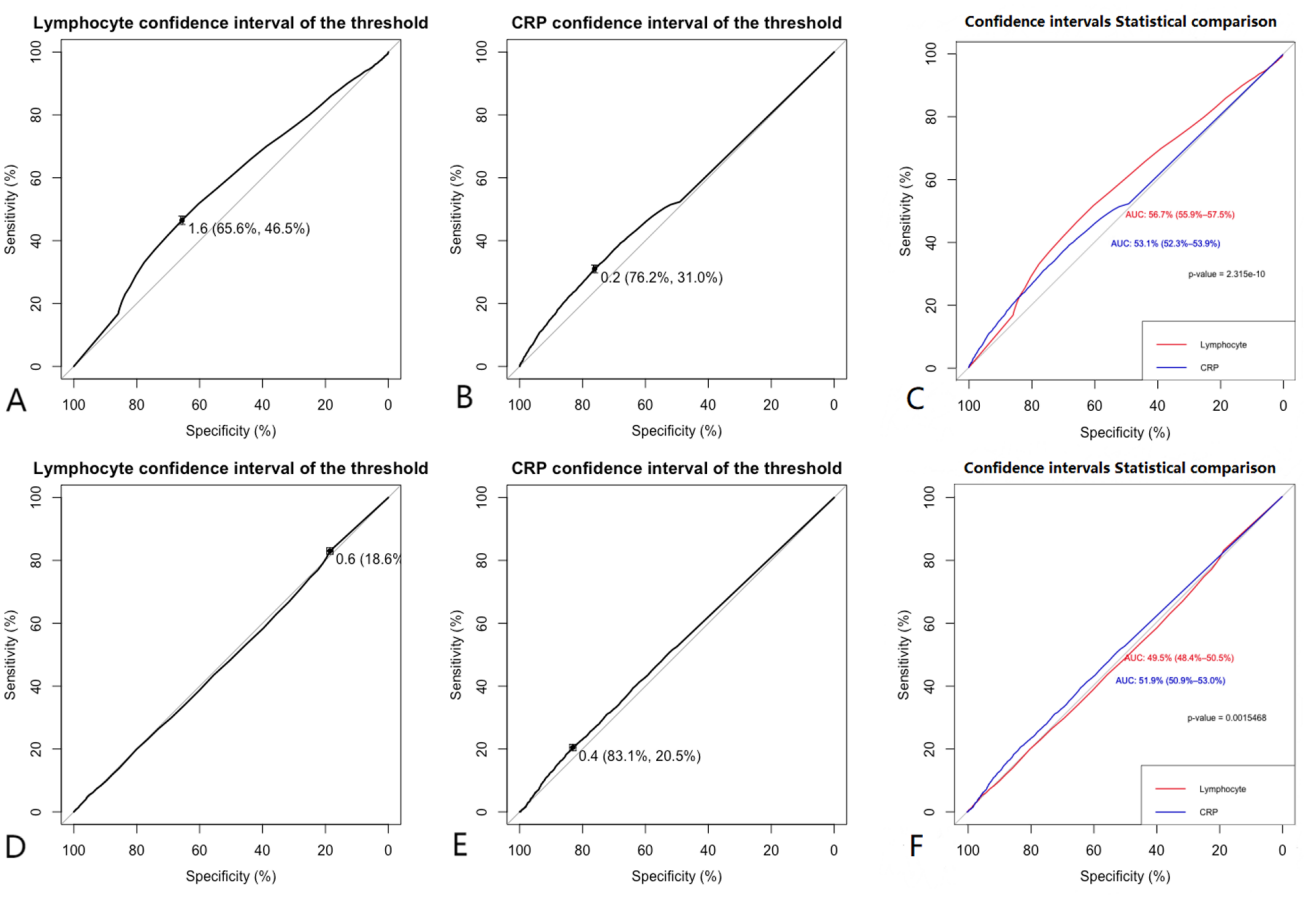
Clinical model. **(A,B)** Show that before matching the ROC curves for Lymphocyte (AUC = 0.567), and CRP (AUC = 0.531), **(C)** indicates that before matching, the diagnostic efficacy of lymphocyte count is higher than CRP (*P* < 0.001). **(D,E)** Show that after matching the ROC curves for Lymphocyte (AUC = 0.495), and CRP (AUC = 0.519), **(F)** indicates that after matching, the diagnostic efficacy of lymphocyte count is lower than CRP (*P* < 0.001). Comparison of ROC curves before and after PSM for lymphocyte count and CRP showed that the diagnostic performance was improved after matching.

**Figure 5:**
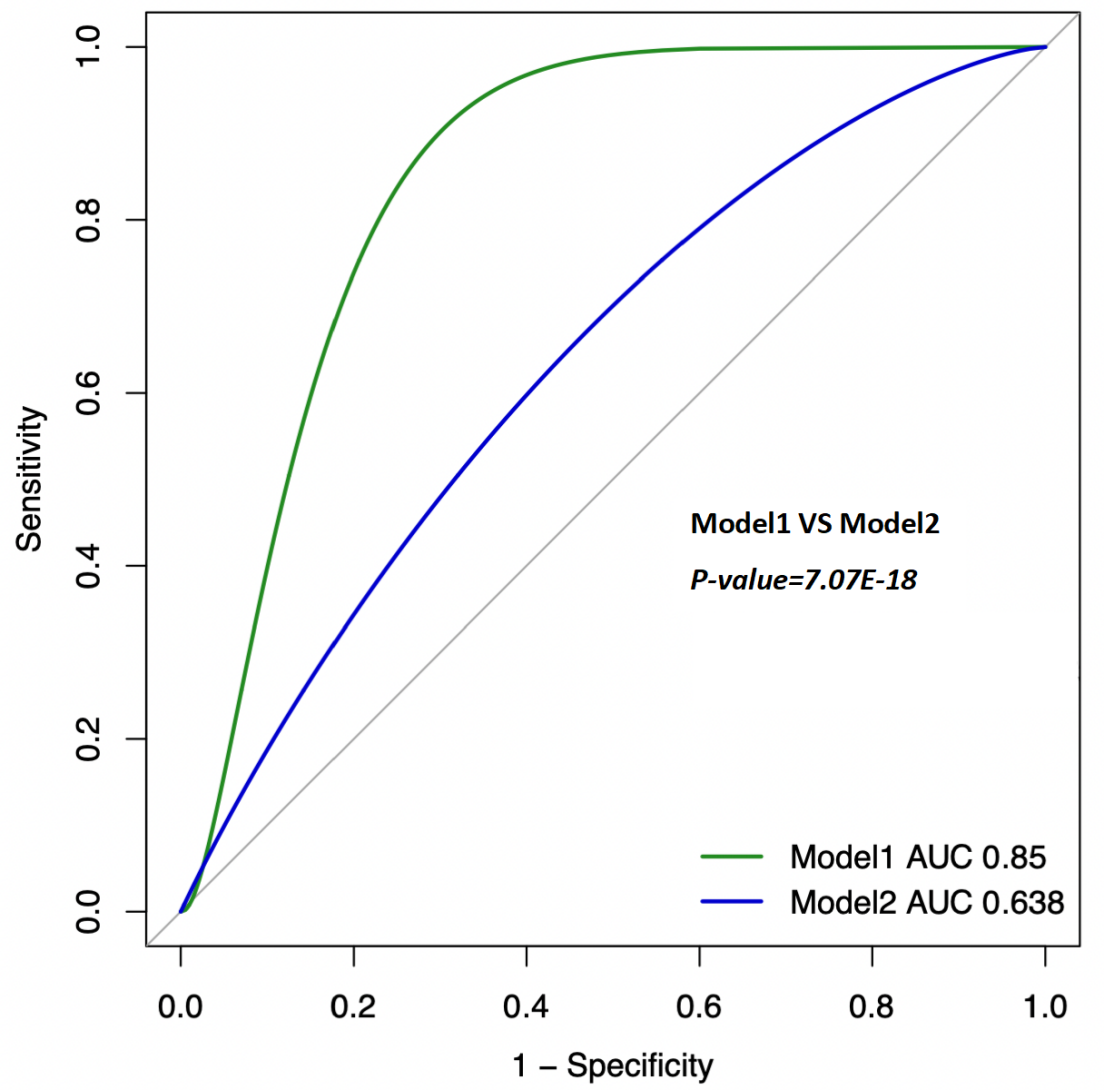
Clinical model (combine). The ROC curves for Model 1 (Lymphocyte + CRP, after PSM); AUC = 0.85 (95% CI 0.819–0.881). The ROC curves for Model 2 (Lymphocyte + CRP, Before PSM); AUC = 0.638 (95% CI 0.6–0.679). PSM: Propensity Score Matching. The ROC curve of the combination of CRP and lymphocyte count showed a moderate diagnostic value for CAD, with improved accuracy after PSM.

Lymphocyte count and C-reactive protein (CRP) were identified as key variables associated with the onset and progression of coronary artery disease (CAD), warranting further evaluation of their diagnostic and prognostic capabilities. Initially, the cutoff value for lymphocyte count was set at 1.6 × 10^9^/L, yielding a sensitivity of 46.5%, specificity of 65.6%, and an area under the curve (AUC) of 56.7% (*P* < 0.05). The cutoff for CRP was set at 0.2 mg/L, resulting in a sensitivity of 31.0%, specificity of 76.2%, and an AUC of 53.1% (*P* < 0.05). After propensity score matching (PSM), the optimal cutoff for lymphocyte count was adjusted to 0.6 × 10^9^/L, showing a sensitivity of 83.2%, specificity of 18.6%, and an AUC of 49.5% (*P* < 0.05). The adjusted CRP cutoff was 0.4 mg/L, yielding a sensitivity of 20.5%, specificity of 83.1%, and an AUC of 51.9% (*P* < 0.05) (see Figure 4 for details). Model 1 (CRP + lymphocyte count) after matching achieved an AUC of 85% (P < 0.05), while Model 2 (CRP + lymphocyte count) before matching recorded an AUC of 63.8% (*P* < 0.05) (see Figure 5 for details). The analysis confirms that both lymphocyte count and CRP have substantial diagnostic significance for CAD, particularly when lymphocyte count exceeds 1.6 × 10^9^/L and CRP exceeds 0.2 mg/L.

### 3.7 Survival analysis

**Figure 6:**
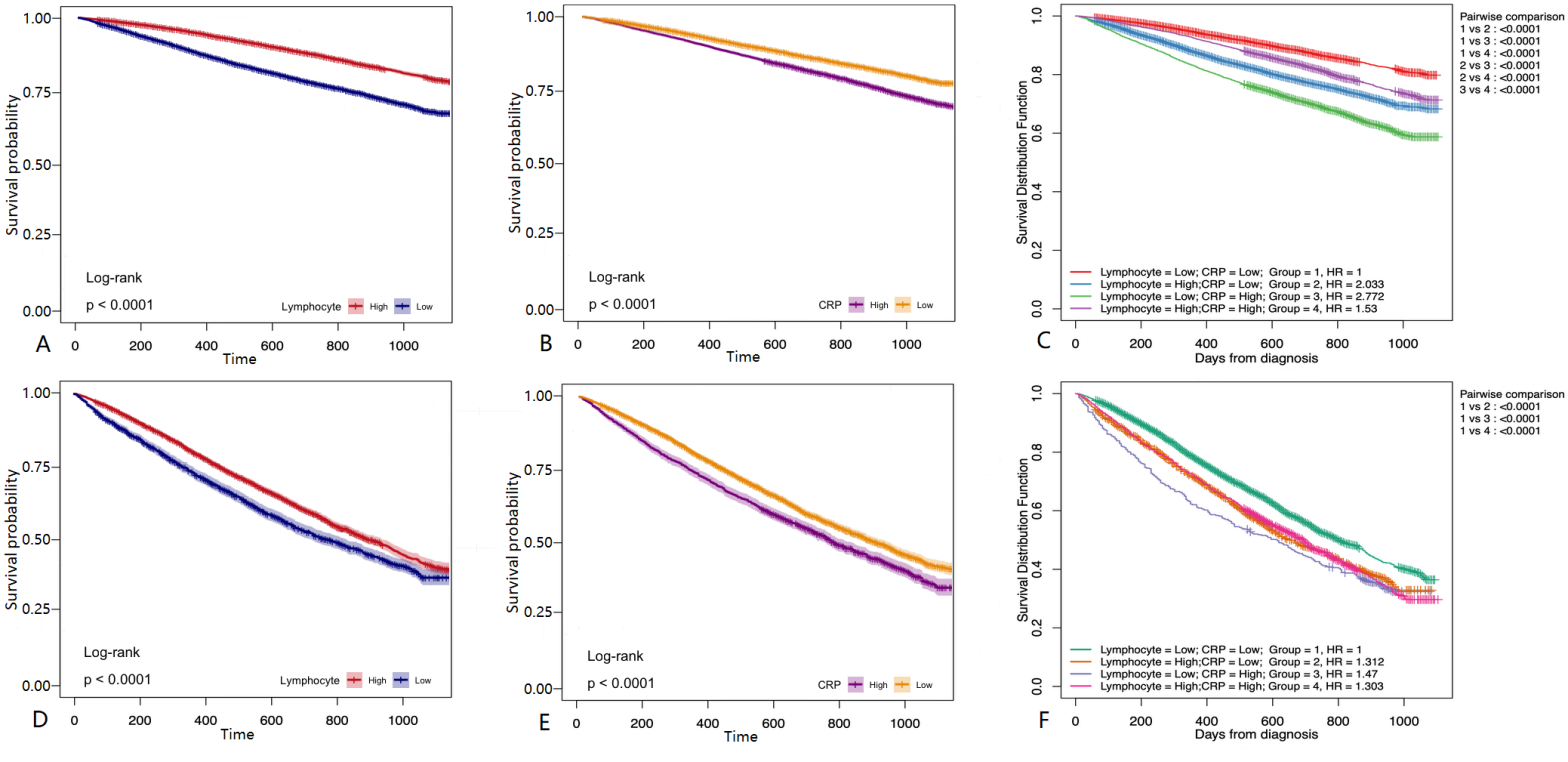
Survival analysis. **(A)** Before PSM matching, The follow-up of patients with different Lymphocyte group for 36 months. **(B)** Before PSM, The follow-up of patients with different CRP group for 36 months. **(C)** Before PSM matching, the follow-up of patients with different Lymphocyte + CRP group for 36 months **(D)** after PSM matching, The follow-up of patients with different Lymphocyte group for 36 months. **(E)** After PSM matching, The follow-up of patients with different CRP group for 36 months. **(F)** After PSM matching, The follow-up of patients with different Lymphocyte + CRP group for 36 months. Kaplan-Meier survival curves showed that patients with high CRP levels and low lymphocyte counts had a higher incidence of MACE, emphasizing the prognostic significance of these biomarkers.

In univariate survival analyses, lymphocyte counts ≤1.6 × 10^9^/L were identified as a risk factor for coronary artery disease (CAD) before propensity score matching (PSM). After PSM, lymphocyte counts ≤0.6 × 10^9^/L continued to be a risk factor. Before PSM, C-reactive protein (CRP) levels >0.2 mg/L were consistently associated with an increased risk of CAD, and this association persisted with CRP levels >0.4 mg/L after PSM. Following a three-year follow-up period, higher lymphocyte counts and lower CRP levels were associated with a reduced risk of major adverse cardiovascular events (MACE) both before and after matching (*P* < 0.001) (Figures 6A,B,D,E). In the combined survival analysis, groups with low lymphocyte counts and low CRP levels served as the reference. Initially, the odds ratio (OR) for participants with high lymphocyte counts and low CRP levels was 2.033 (*P* < 0.001). The ORs were 2.772 for those with low lymphocyte counts and high CRP levels, and 1.53 for those with high levels of both lymphocytes and CRP (*P* < 0.001). Post-matching, the OR adjusted to 1.53 for the first group, 1.312 for those with high lymphocyte counts and low CRP levels, 1.47 for those with low lymphocyte counts and high CRP levels, and 1.303 for those with high levels of both indicators (*P* < 0.001) (Figures 6C,F). Notably, high CRP levels were associated with a more significant impact on the occurrence of MACE.

## 4 Discussion

In this study, the causal relationship between leukocyte subtypes, CRP and CAD was first analyzed using TSMR. The study included 5,467 patients with confirmed CAD and 41,197 controls. Post PSM analysis, logistic regression, Lasso regression, and RF algorithms demonstrated strong associations between lymphocyte counts, CRP levels, and the development of CAD. ROC curves determined the optimal cutoff values for lymphocyte count (>1.6 × 10^9^/L) and CRP levels (<0.2 mg/L) in the study population, while also evaluating their diagnostic sensitivity and specificity for CAD, both individually and combined. Survival analyses, considering various lymphocyte counts and CRP levels, indicated that lower lymphocyte counts and higher CRP levels were linked to an increased incidence of MACE, with a significant contribution from CRP levels.

CRP is an acute-phase reactant protein that reflects the body's inflammatory state (33). Recent evidence increasingly suggests that inflammation significantly contributes to the development of atherosclerosis and CAD (34–36). Beyond its role in forming atherosclerotic plaques, inflammation also contributes to their instability and rupture (5). CRP influences endothelial activation and dysfunction by modifying endothelial vasoreactivity, primarily through a reduction in endothelial nitric oxide synthase (eNOS) activity, which may heighten the risk of atherothrombosis, hypertension, and CAD (37). Elevated CRP levels (below 10 mg/L) are generally associated with an increased cardiovascular risk (5, 38, 39). Our findings align with this perspective, suggesting that high CRP levels may signal ongoing inflammatory activity within the arteries, potentially leading to plaque instability and an elevated risk of MACE. An eight-year follow-up study by Ridker et al*.* (40)*.* involving 27,939 female patients showed a correlation between CRP levels and the incidence of cardiovascular events, indicating CRP's association with CAD onset in women. Cushman et al*.* (41)*.* measured baseline CRP levels in 3,971 older adults without prior cardiovascular disease and conducted a ten-year follow-up. After adjusting for confounding factors, they found that individuals with CRP levels above 3 mg/L had a relative risk (RR) of 1.45 for CAD compared to those with levels above 1 mg/L. In a 1:1 case-control study of 420 CAD patients by Liu et al*.* (42), individuals in the acute coronary syndrome (ACS) group exhibited significantly higher CRP levels compared to controls. A notably higher incidence of MACE was observed among patients with elevated CRP levels. Even after adjusting for baseline confounders, CRP levels remained an independent predictor of MACE, with higher values linked to significantly greater all-cause mortality and myocardial infarction rates during the follow-up. In a five-year study of 3,802 participants, Zhuang Qian and colleagues (43) found that those with hs-CRP levels ≥1.08 mg/L faced a higher risk of developing CAD than those with hs-CRP levels <1.08 mg/L. However, subsequent MR analysis did not establish a significant causal relationship between hs-CRP and CAD. The difference in data sources from GWAS in our study might explain the divergent conclusions, attributable to variances in sample sources.

Lymphocytes play a crucial role in the human immune system, and alterations in their number and functionality are associated with various pathological conditions. Reduced lymphocyte levels can suggest chronic inflammation and immunosuppression, both involved in the pathophysiological processes of CAD (44). Atherosclerosis, a chronic inflammatory disease, increasingly recognizes the significance of lymphocytes. A decrease in lymphocyte count may reflect a reduced ability of the immune system to manage inflammatory responses, thus expediting the development and progression of coronary artery lesions. The pathological mechanisms involving lymphocytes in CAD are intricate, with different lymphocyte subtypes playing specific roles (44, 45). Zhang et al*.* (46)*.* demonstrated that T lymphocytes facilitate atherosclerosis development by producing pro-inflammatory cytokines and adhesion molecules, prompting monocyte migration to the subendothelial layer. Additionally, numerous studies (47–49) have underscored the protective roles of natural and induced regulatory T cells (nTreg and iTreg) in atherosclerosis, possibly by diminishing the antigen-presenting activity of dendritic cells, thereby moderating both innate and adaptive immune cell activation. In mouse-based research, Kayw (50) and colleagues showed that B lymphocyte subtypes B1 and B2 jointly affect atherosclerosis development. B1 cells produce IgM antibodies that primarily recognize oxidized LDL (ox-LDL), conferring a protective effect against atherosclerosis, whereas B2 cells produce IgG and IgE, linked to the promotion of the disease. Acanfora et al*.* (51)*.* demonstrated that lymphocyte levels are significantly correlated with the prognosis of patients with acute coronary syndrome, with low levels associated with an elevated risk of adverse cardiovascular events. Anoop Dinesh Shah et al*.* (52). conducted a cohort study revealing that low lymphocyte levels (≤ 1.45 × 10^9^/L) were significantly associated with CAD mortality within the first six months (OR = 2.25, 95% CI 1.90–2.67), with a weaker correlation noted thereafter.

The established role of CRP as an inflammatory marker in CAD is well recognized, and the significance of lymphocytes in CAD is increasingly acknowledged. Despite this, the precise mechanisms and practical applications of this relationship warrant further investigation. The current study enhances the existing evidence by demonstrating a correlation between CRP levels and lymphocyte counts in CAD patients. Both peripheral blood lymphocyte counts and CRP levels may serve as indicators for assessing immune status and inflammation in these patients. Regular monitoring of CRP and lymphocyte levels in high-risk populations could enable early detection of individuals susceptible to CAD and facilitate timely preventative or therapeutic interventions. However, additional research is necessary to confirm the prognostic value of CRP and lymphocyte counts in CAD and to guide clinical decision-making. Future studies should explore the underlying mechanisms of CRP and lymphocyte fluctuations in CAD, evaluate their potential as therapeutic targets, and develop tailored intervention strategies to enhance the prevention and management of cardiovascular diseases.

Our study innovatively integrated MR with a comprehensive real-world analysis, which validated prior research conclusions and enhanced the reliability of these findings. However, it is critical to acknowledge the inherent limitations of this study. Being a single-center observational study, the generalizability of the results may be limited. Moreover, as a retrospective study, our research could be influenced by certain confounding factors and selection bias. The study also did not control for all possible confounders such as other chronic diseases, medication use, and lifestyle factors, which might affect CRP and lymphocyte levels, as well as the risk of CAD. In future research, we aim to expand the sample size and strive to include multi-center data. Additionally, more data and extended follow-up are necessary to predict risk factors and prognosis for CAD patients accurately. Therefore, it is essential to conduct further randomized controlled trials to validate our findings.

## 5 Conclusion

There is a causal relationship between lymphocytes, CRP and CAD. The combined assessment of CRP and lymphocytes offers diagnostic value for CAD. Furthermore, high CRP levels coupled with low lymphocyte counts are associated with a poor prognosis.

## Data Availability

The raw data supporting the conclusions of this article will be made available by the authors, without undue reservation.
